# Automated single cell isolation from suspension with computer vision

**DOI:** 10.1038/srep20375

**Published:** 2016-02-09

**Authors:** Rita Ungai-Salánki, Tamás Gerecsei, Péter Fürjes, Norbert Orgovan, Noémi Sándor, Eszter Holczer, Robert Horvath, Bálint Szabó

**Affiliations:** 1 Doctoral School of Molecular- and Nanotechnologies, University of Pannonia, Veszprém, Hungary; 2 Nanobiosensorics Group, Institute of Technical Physics and Materials Science, Centre for Energy Research, Hung. Acad. Sci., Budapest, Hungary; 3 Department of Biological Physics, Eötvös University, Pázmány Péter sétány 1A, Budapest, H-1117 Hungary; 4 MEMS Lab, Institute of Technical Physics and Materials Science, Centre for Energy Research, Hung. Acad. Sci., Budapest, Hungary; 5 MTA-ELTE Immunology Research Group, Budapest, Hungary; 6 CellSorter Company for Innovations, Budapest, Hungary

## Abstract

Current robots can manipulate only surface-attached cells seriously limiting the fields of their application for single cell handling. We developed a computer vision-based robot applying a motorized microscope and micropipette to recognize and gently isolate intact individual cells for subsequent analysis, e.g., DNA/RNA sequencing in 1–2 nanoliters from a thin (~100 μm) layer of cell suspension. It can retrieve rare cells, needs minimal sample preparation, and can be applied for virtually any tissue cell type. Combination of 1 μm positioning precision, adaptive cell targeting and below 1 nl liquid handling precision resulted in an unprecedented accuracy and efficiency in robotic single cell isolation. Single cells were injected either into the wells of a miniature plate with a sorting speed of 3 cells/min or into standard PCR tubes with 2 cells/min. We could isolate labeled cells also from dense cultures containing ~1,000 times more unlabeled cells by the successive application of the sorting process. We compared the efficiency of our method to that of single cell entrapment in microwells and subsequent sorting with the automated micropipette: the recovery rate of single cells was greatly improved.

We built a semi-automated device from affordable commercial components, which is able to complete a delicate task currently carried out by skillful experts trained to do difficult manipulations on a microscope. Our system is controlled by computer vision bearing the potential for exploiting advanced image processing algorithms, including artificial intelligence to select specific cells.

Single cell DNA and RNA analysis utilizing next generation sequencing^1^ is a promising tool of molecular cell biology. It is already applicable for cancer research^2^, and can answer some fundamental questions of cell biology^3^. Manual single cell isolation for DNA/RNA sequencing from a suspension with a mouth micropipette is a precise but very low throughput method requiring a well-trained expert^4^. Flow cytometry-based fluorescence-activated cell sorters (FACS) have been used for several decades, and became the default technique for sorting cells one-by-one^5^,^6^. Modern FACS machines can have several channels to detect fluorescence, and a sort rate of 10,000 cells per second or more. Development of on-chip μFACS devices^7^,^8^ opens new perspectives. However, if the number of target cells is very low or single cells have to be isolated in different vessels FACS technology becomes cumbersome. Laser-capture microdissection^9^ can isolate selected cells even from a tissue slice. Related techniques, e.g., laser-enabled analysis and processing (LEAP)^10^ emerged for more specialized applications. Nevertheless, high-throughput single cell isolation has not been realized with such laser-mediated techniques up to now. Integrated fluidic circuits^11^ can trap and isolate single cells with a relatively high throughput, e.g., into 96-well plates^12^. However, the high level of integration allows less control for the user in the specific study, and optimized microfluidics can be highly sensitive to cell size and rigidity. Fluorescent imaging-based cell selection and subsequent sequencing is expected to give far more information on the functional aspects of the molecular phenotype and genotype of single cells. Existing robots can detect and isolate surface-attached cells only^13^,^14^,^15^,^16^,^17^,^18^,^19^. The strength of cell adhesion has to be kept in a certain regime. Although naturally adherent cells can be spontaneously immobilized on a bare plastic or glass surface, the adhesion force needs to be tuned either biochemically or by surface modifications optimized for the cell type^15^,^16^. Otherwise the too strongly adhered cells are picked up at an expense of damaging the cell. Naturally non-adherent cells are artificially perturbed, when forced to adhere to a surface, which may alter their gene expression profile. Cells trapped in cell-size specific microwells also tend to adhere too strongly to the surface and either get damaged when picked up with a high force or lost when the picking force is insufficient. Fluid flow through a microcavity array can mechanically trap single cells enabling automated cell isolation^13^. An advanced version^20^ of the microcavity array applying a punch needle to isolate cells through the membrane has been introduced recently. However, microcavity arrays interfere with imaging, which can be a drawback if the analysis needs a high-resolution image of entire cells. In addition, the production of such specialized microstructures needs advanced micromachining technology hindering their widespread application. Cell encapsulation in nano- or picoliter-scale droplets^18^,^21^,^22^ is a promising route for single cell manipulations in water-oil-based two-phase microfluidic systems. Nonetheless, it could not gain extensive use, probably due to the technical challenges of operating complex microfluidic chips.

A robot with computer vision-based feedback and closed-loop process control was demonstrated to sort single cells^19^. This system also used initially immobilized cells, and bright-field illumination was critically needed for the closed-loop motion control of the micropipette. In a dense culture such cell capture is impractical as the targeted cell will be mixed up with its neighbors.

Our method can readily isolate single live cells even from a very dense culture without immobilizing cells on a surface. Assuming that the micropipette aperture is chosen accordingly to the cell size, the technique can be applied to virtually any tissue cell type without a sensitivity to cell rigidity or adherence. Although the current study is limited to the isolation of mammalian cells, insect, plant and yeast cells can also be isolated by a micropipette as it is carried out manually in many laboratories.

## Results

### Developing the technique of single cell isolation from a thin layer of suspension

**Figure 1:**
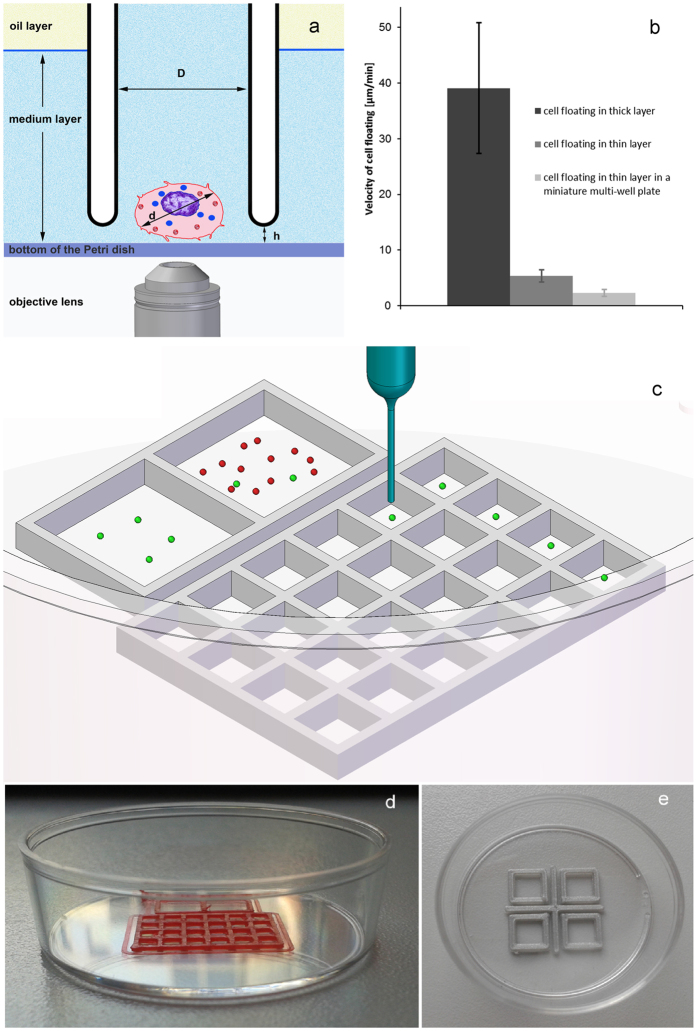
Automated micropipette for single cell isolation from a thin layer of suspension. Panel (**a**) shows the concept of cell sorting. Cells are detected by computer vision using the objective lens of an inverted microscope. Cell suspension is confined into a thin ~100 μm layer of culture medium or buffer covered with oil to avoid the convection-driven floating of cells. The glass micropipette with an inner diameter of D = 30 μm approaches the surface of the dish to a distance of h = 5 μm. Targeted cell is picked up by a slight vacuum connected to the micropipette and controlled by a high speed fluid valve. Inhibitory effect of cell confinement into a thin layer on cell floating is shown in (**b**). Wells of the miniature plate (shown in (**c–e**)) printed into the Petri dish further decreased convection. Panel **c** presents the schematics of the experiment. Initial suspension was kept in a larger, 5 × 5 mm^2^ square. Green and red dots represent cells to be isolated and cells not needed, respectively. When using a sparse suspension (Fig. 2), selected single cells could be directly moved from this square to the other large square or into smaller, 2 × 2 mm^2^ squares in the same dish or into PCR tubes (not shown). Photos of the miniature multiwell plates printed into 35 mm plastic Petri dishes with a resolution of 0.2 mm using a commercial 3D printer (Ultimaker) are shown in panel (**d–e)**. (**d**) side view of a 24-well plate with 2 × 2 mm^2^ wells beside the two larger (5 × 5 mm^2^) squares inside the Petri dish. Four 5 × 5 mm^2^ wells (shown in (**e**)) were used when isolating single cells from a dense culture in successive pickup and deposition steps. We printed the structures with a height of 0.5 or 1.0 mm.

To isolate single cells from suspension we used a motorized microscope and micropipette controlled by computer vision. Combination of 1 μm positioning precision, adaptive cell targeting, and below 1 nl liquid handling precision resulted in an unprecedented accuracy and efficiency in robotic single cell isolation. To minimize fluid convection, being the major effect driving passive cell movements in suspension, cells were kept in a thin (97 ± 10 μm) layer of buffer or culture medium covered with oil (Fig. 1a,b).

We applied a commercial 3D printer to build miniature multi-well plates from polylactic acid (PLA) with a height of 0.5 or 1.0 mm into the Petri dish. Cell monolayers were generated in the larger, 5 × 5 mm^2^ squares (Fig. 1c–e) with a density up to 2,000 cells/mm^2^ by injecting 5–10 μl cell suspension into the thin medium layer under the oil using a manual laboratory pipette. All the cells could be retained inside the square up to 10,000 cells, and more than 99% of cells remained inside after injecting 50,000 cells into the square proving the ability of the low (1 mm) walls to keep cells inside. A representative high density culture is shown in Supplementary Fig. S1-2.

We scanned in the region of interest by capturing a mosaic image covering the whole or a part of the square. We selected cells for isolation in fluorescent imaging mode to demonstrate the ability of the technique to sort cells on a molecular basis. Fluorescent cells were detected by computer vision using a local variance method for image segmentation^15^ implemented in the CellSorter software. For optimizing cell selection the operator could tune the detection parameters including minimum and maximum brightness and size of cells to be selected, detection sensitivity, and image noise level. This human feedback taking normally a few seconds (less than a minute) enabled the algorithm to recognize labeled cells with high efficiency even if the staining of cells or imaging parameters were very different from previous experiments. External image segmentation algorithms can also be used in concert with the sorting software for specialized cell selection tasks. We could readily detect cells with the Analyze Particle function of the open source ImageJ software, and tune the detection by changing the parameters. It was possible to detect only circular or elongated cells. After selecting cells for isolation we ran the sorting process. Real-time vision-based feedback for cell targeting is disadvantageous as the exposition time has to be minimized in fluorescent mode to avoid photo-bleaching and/or photo-damaging of cells. Thus, instead of using a closed-loop feedback for locating and picking cells potentially displaced from their original position, we applied adaptive cell targeting. Initial cell coordinates saved when scanning the region of interest were corrected by capturing a single new image right before picking the next cell.

Our method allowed the high-throughput scanning and analysis of cell suspensions: ~1,000 or ~10,000 cells/min in case of a sparse or dense culture, respectively (Supplementary Fig. S3). Isolation of the selected cells is slower: 2–3 and ~1 cells/min for sparse and dense cultures, respectively. However, when a small population needs to be isolated from a large number of cells the technique is very efficient. (The culture is considered to be dense if the typical distance of neighboring cells is lower than the sorting resolution detailed in the next paragraph). The method is scalable up to millions of cells to be analyzed in a single run by increasing the scanned area.

### Application of the new technique

#### Single cell isolation from a sparse suspension

Single cells could be picked up in a volume of 1.4 ± 0.6 nl without removing neighboring cells. Using a micropipette with an inner diameter (I.D.) of 30 μm the spatial resolution of the technique was 34 ± 4 μm, i.e., cells farther from the targeted cell than this distance were not removed. Single cells were injected either into the wells of the miniature plate with a sorting speed of 3 cells/min or into standard PCR tubes with 2 cells/min. Sorting process did not affect the viability of cells.

#### Single cell entrapment in microwells and subsequent sorting

**Figure 2:**
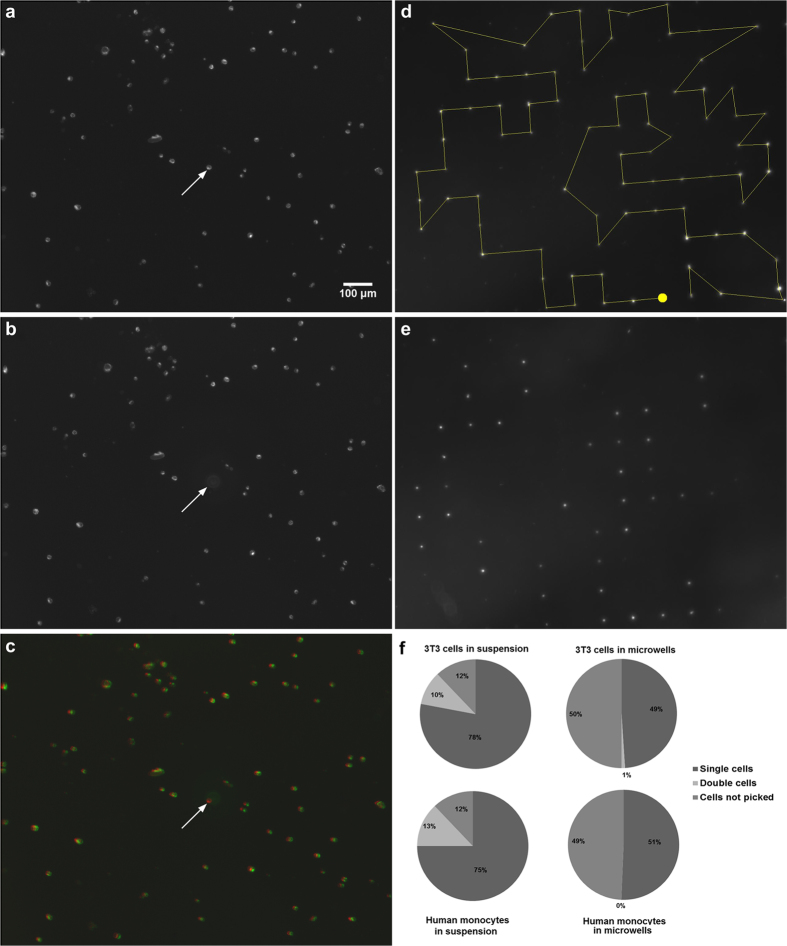
Single cell isolation from a sparse suspension. Panels (**a–c**) show the pick-up process of a single cell from a sparse fluorescent suspension. Arrow points to the location of the selected cell visible in **(a)** before picking it with the micropipette. (**b**) shows the same region after picking the cell. Tip of the I.D. 30 μm micropipette is visible in (**b**) at the location of the removed cell. (**c**) shows a combined image of (**a**,**b**) converted to red and green, respectively. Displacement of cells can be observed as green cells do not perfectly overlap with red ones. Green image of the selected cell is missing but all other cells remained in the dish. We compared our results to the method of single cell trapping in PDMS microwells and subsequent sorting (**d,e)** with a micropipette. Path of the I.D. 70 μm micropipette visiting all detected cells is shown in **(d)**. Yellow dot marks the first cell to be picked. A significant ratio of cells (shown in (**e**)) adhered too strongly into the PDMS wells and thus could not be picked up. Comparison of the efficiency of the two techniques is summarized in (**f**). Ratio of successful single cell isolation was improved from about 50% to above 75% when using the new technique eliminating cell adhesion (Supplementary Table S1).

We compared the efficiency of our method to that of single cell entrapment in microwells^23^ and subsequent sorting with the automated micropipette (Fig. 2). As the spatial resolution of the I.D. 70 μm micropipette normally used to pick surface attached cells is ~70 μm^15^, we applied PDMS microwells arranged to a grid with a lattice constant of 100 μm (Supplementary Fig. 4) to avoid picking unselected cells from the neighboring wells.

The recovery rate of single cells was greatly improved when sorting from a thin layer of suspension instead of using microwells. In case of microwells 98–99% of cells were not trapped, i.e., they were lost in washing steps. Although this ratio can be improved by decreasing the distance between microwells, the number of lost cells is always significant. Furthermore, the sorting efficiency of the micropipette is reduced when applying closely packed microwells. Efficiency of filling a microwell with a cell was 25 ± 8% and 54 ± 7% for 3T3 cells and monocytes, respectively. ~50% of the trapped and selected single cells could be successfully isolated using microwells. This ratio was improved to ~75% when sorting from a thin layer of suspension (Fig. 2, Supplementary Table S1). More importantly, the number of cells lost in the preparation steps of our new technique is practically zero.

#### Sorting from a dense suspension

**Figure 3:**
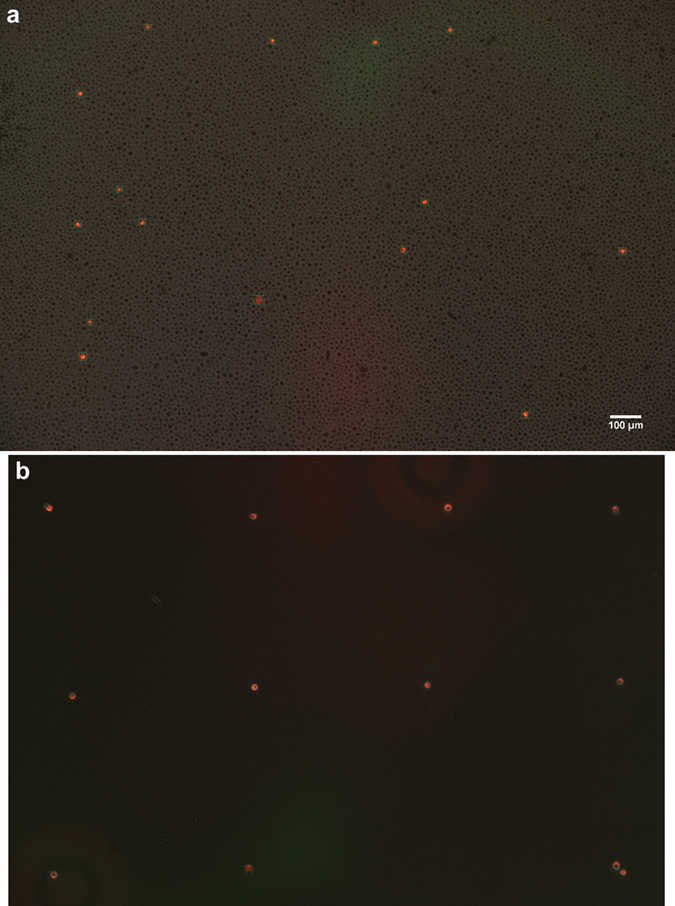
Single cell isolation from a dense suspension. Our method could isolate single labeled cells from dense cultures containing ~1,000 times more unlabeled cells. Panel (**a**) shows a combined picture of the red fluorescent and the greyscale phase contrast images with 15 cells in green frames detected by the software and ~17,000 unlabeled cells. By repeating the sorting process in 3 successive steps we could isolate most of the labeled single cells into a new well. Panel (**b**) (similarly to (**a**)) is a combined picture of the fluorescent and phase contrast images with the isolated 12 fluorescent cells deposited 500 μm from each other in a grid. Final culture contains zero unlabeled cells. Last 2 cells were injected to the same location as the deposition of the #11 cell did not succeed when tried at its planned location.

When sorting from a dense culture (Fig. 3), unlabeled cells were also picked up from the proximity of the targeted single cell. We used a Petri dish with four 3D-printed 5 × 5 mm^2^ squares (Fig. 1e), and ran additional sorting cycles to get rid of the unlabeled cells^4^. Deposited cells were scanned and sorted again into a new square in the same Petri dish. After three successive runs the procedure resulted in a clean cell population without any unlabeled cells. When the initial culture contained 1,000 times more unlabeled cells than labeled ones, the device isolated 25 ± 2 unlabeled cells per labeled cell in the first step. In the next step the number of the remaining unlabeled cells decreased to 1.8 ± 0.8 per labeled cell. In the third run only the labeled cells were transferred to the new square.

## Discussion

We developed a computer vision-based robot applying a motorized microscope and micropipette to recognize and gently isolate intact individual cells for subsequent analysis, e.g., DNA/RNA sequencing in 1–2 nanoliters from a thin (~100 μm) layer of cell suspension. It can retrieve rare cells, needs minimal sample preparation, and can be applied for virtually any tissue cell type. Combination of 1 μm positioning precision, adaptive cell targeting and below 1 nl liquid handling precision resulted in an unprecedented accuracy and efficiency in robotic single cell isolation.

To enable the CellSorter system^15^,^16^ to isolate suspended single cells we introduced the following innovations and improvements:

1. Adaptive cell targeting.
2. 1 nl liquid handling precision by optimizing the vacuum value and the micropipette diameter together, and applying precise ambient pressure for stopping the flow.
3. To minimize fluid convection cells were kept in a thin layer of buffer covered with oil.
4. We applied a 3D printer to build miniature multi-well plates into the Petri dish to confine the initial culture and isolated cells into different wells.

We isolated fluorescent cells also from dense cultures containing ~1,000 times more unlabeled cells by the successive application of the sorting process. Comparing the efficiency of our method to that of single cell entrapment in microwells and subsequent sorting we conclude that the recovery rate of single cells was greatly improved. We expect that image-based automated single cell manipulation will become an everyday technique of molecular cell biology. Cell selection can be based on complex and specific image analysis; even time-lapse imaging can be used to select appropriate cells for isolation.

## Methods

### Cell culture of human monocytes

Peripheral blood mononuclear cells (PBMCs) were isolated from buffy coat obtained from healthy donors and provided by the Hungarian National Blood Transfusion Service by density gradient centrifugation on Ficoll-Paque (GE Healthcare). Monocytes were isolated by negative magnetic separation using the Miltenyi Monocyte Isolation kit II (Miltenyi) according to the manufacturer’s instructions. Informed consent was provided for the use of blood samples according to the Declaration of Helsinki. Cells were cultivated in Roswell Park Memorial Institute (RPMI) medium supplemented with 10% fetal calf serum (FCS) (37 °C, 5% CO_2_ atmosphere). Where indicated, monocytes were loaded with 0.5 μM carboxyfluorescein succinimidyl ester (CFSE) fluorescent dye (Molecular Probes). Cells were mixed with appropriate dye concentration and incubated for 10 min at room temperature in dark. After that they were washed 3 times with phosphate buffered saline (PBS) containing 5% FCS to remove excess dye. The loaded monocytes were immediately used in the experiments.

### 3T3 cell culture

Mouse embryonic fibroblasts (ATCC; CCL-92) were grown in MEM supplemented with 10% FCS (Sigma), according to the guidelines of the ATCC Cell Biology Collection. A subpopulation of the cells was stained with the lipophilic fluorescent dye, DiI (10 μM, 30 minutes at 37 °C, Invitrogen). Before sorting, 3T3 cells were treated with 1x trypsin-EDTA solution (Gibco, 25300) for 6 min, at 37 °C, then the cell suspension was centrifuged at 300 g for 2 min.

### Fabrication of PDMS microwells

We used wells with a diameter of 15 and 20 μm fitted to the size of human monocytes and 3T3 cells, respectively. The lithographic mask of the microwell array was manufactured by a Heidelberg DWL 66 + laser pattern generator. Final microwell arrays were developed by soft lithography^24^ in polydimethylsiloxane (PDMS, Dow Corning Sylgard 184) applying SU-8 pattern as the molding replica. SU-8 (MicroChem) is an epoxy based negative photoresist, conventionally used for creating microstructures with high aspect ratio^25^,^26^. A silicon substrate was spin-coated with SU-8 3025 photoresist using a Brewer Science Cee 200CBX coat-bake system at 3000 rpm, and exposed to UV light with a Süss Mikrotech MA6 mask aligner. Dose of the H-line (405 nm) exposure was ~300 mJ/cm^2^. We applied 1-methoxy-2-propyl acetate solution to remove the unexposed resist. Geometric parameters of the resulted microstructures were characterized by stylus profiler (Bruker Dektak XT). Quality of the master replica was further verified by a scanning electron microscope (SEM, Zeiss LEO XB1540) with low acceleration voltages (V_acc_ = 2 keV) at 10^−7^mbar chamber vacuum according to the resistive characteristics of the sample. The PDMS elastomer and the curing agent were mixed in 10:1 ratio and molded onto the photoresist replica, polymerized at room temperature for two days, and peeled off from the molding form. Final PDMS structures were characterized by SEM (Supplementary Fig. S4)

### Trapping single cells in PDMS microwells

After the 10 × 10 mm^2^ PDMS plate with microwells was inverted and laid into a 35 mm Petri dish (Greiner) containing cell culture medium, we placed it into the cell culture incubator (37 °C, 5% CO_2_) for ~ 2 hours in order to fill the wells with culture medium^23^. Then the medium was removed from the dish and with the exception of the microstructure all sides of the PDMS plate were wiped. Cells were counted with a hemocytometer and 200–400,000 cells were centrifuged at 300 g for 6 min. After centrifugation, the supernatant was removed and the pellet was resuspended in 100 μl medium and cells were pipetted onto the PDMS structure, and incubated for 30 min at 37 °C. Floating cells were removed from the PDMS by gently washing the PDMS with a pipette. Lastly, the PDMS plate was pressed to the bottom of a new Petri dish and covered with medium.

### Printing miniature multiwell plates into the Petri dish

We applied a commercial 3D printer (Ultimaker) slightly modified to enable printing into 35 mm Petri dishes. We built miniature multi-well plates from polylactic acid (PLA) with a height of 0.5 or 1.0 mm into the Petri dish either into untreated hydrophobic or cell culture Petri dishes (Greiner) in order to keep suspended cells in a specified area of the dish. We used 2 structures shown in Fig. 1. One with two larger (5 × 5 mm^2^) squares and 24 smaller (2 × 2 mm^2^) squares for isolating single cells from a sparse culture (Fig. 1d). For the successive single cell isolation process from dense culture we used four 5 × 5 mm^2^ squares (Fig. 1e).

### Creating a thin layer of cell suspension in the Petri dish

We used untreated hydrophobic plastic Petri dishes (Greiner) for the 3T3 cells. Hydrophilic tissue culture dishes (for monocytes) were coated with 1 mg/ml PLL-g-PEG (SuSoS) for 30 min, at room temperature to inhibit cell adhesion. After covering the Petri dish with 2 ml culture medium, we layered 1 ml silicon oil (Silicon oil AR 20, Sigma Aldrich) or mineral oil (Sigma Aldrich) onto the culture medium. To thin the layer of the culture medium we removed the excess medium from beneath the oil using a pipette, and injected it into an Eppendorf tube. We estimated the height of the remaining medium layer in the Petri dish by measuring the weight of the removed medium on an analytical balance (Scaltec SBA 32). When sorting from a sparse culture, 2,000 cells in 5–10 μl of culture medium supplemented with 10% FCS were injected into a 5 × 5 mm^2^ square of the miniature plate in the Petri dish. When sorting from a dense culture, 50,000 unlabeled and 50 DiI-labeled 3T3 cells were injected into a 5 × 5 mm^2^ square in a volume of 5–10 μl using a manual laboratory pipette.

### Automated single cell isolation from cell suspension

We used an automated micropipette setup (CellSorter)^15^,^16^ to detect and isolate single cells on a microscope. Briefly, an inverted microscope (Zeiss Axio Observer A1) equipped with a 10x objective lens (EC Plan Neofluar), digital camera (Qimaging Retiga 1300 or Canon EOS 40D or Andor Zyla 5.5 USB 3), motorized stage (Marzhauser Scan IM 130 × 100), focus motor (Marzhauser) was applied to scan a region of interest in the Petri dish in phase contrast or fluorescent mode. A microfluidic system (CellSorter) controlled the flow in the glass micropipette with an inner diameter of 30 μm.

Before picking up the first cell, culture medium was let into the micropipette to avoid the osmotic shock of cells. The tip of the micropipette was positioned in all 3 dimensions with 1 μm precision. We carefully touched the surface of the Petri dish with the tip to precisely calibrate its vertical position. Either a vacuum of −100 Pa or an overpressure of 4,200 Pa were generated in the same syringe (syringe 1) using a syringe pump (New Era, NE-1000)^16^. The pressure in syringe 2 was constantly the same as in the Petri dish, i.e., ambient pressure. After positioning the micropipette above the next cell, and approaching the surface to 5 μm, the vacuum preset in syringe 1 was applied to the micropipette by opening valve 1 for 10 ms. To quickly terminate both the vacuum and the flow in the micropipette after picking the cell, we opened valve 2 connected to syringe 2 with ambient pressure for 1 s. For depositing cells we used the same method but preset overpressure in syringe 1 and applied it for 1 s.

### External image segmentation

External image segmentation algorithms could also be used in concert with the CellSorter sorting software for specialized cell selection tasks. The external algorithm has to retrieve the coordinates of recognized cells, and optionally the dimensions of the bounding rectangle fitted onto the cell and the average brightness of the cell. Data can be transferred via the Windows clipboard or in a .csv file. We tested the method by converting the fluorescent mosaic image to 8-bit greyscale first, then applying the MaxEntropy Threshold, and finally the Analyze Particles function (with the Record starts option checked) of the open source ImageJ^27^, software.

### Adaptive cell targeting

Although fluid convection was minimized in the oil covered thin layer of cell suspension, cells were not perfectly immobilized (Fig. 2c). To achieve perfect cell targeting we corrected the coordinates of displaced cells. A fluorescent image was captured right before picking up the next cell. Software using the same parameters as applied when initially scanning the whole region of interest and detecting cells, located the cells again and corrected the original coordinates of the next cell. Thus, we detected each cell twice by computer vision: first when scanning the whole region of interest, secondly right before targeting the cell. The micropipette was positioned to this corrected location of the cell.

### Optimization of the experimental parameters

In order to maximize single cell sorting efficiency, we optimized the following experimental parameters.

1. Hydrophobicity of the Petri dish to avoid cell adhesion during sorting. Optimal surfaces were the untreated hydrophobic plastic Petri dish and the PLL-g-PEG coated hydrophilic (tissue culture) dish.
2. Vacuum and pressure values applied to the micropipette when picking up and depositing cells, respectively. Optimal vacuum was −100 Pa. Optimal overpressure was 4,200 Pa. Flow in the micropipette was stopped by applying precise 0 Pa to the micropipette.
3. Diameter of the glass micropipette. Larger micropipettes picked up more cells in a larger volume, whereas cells tended to adhere inside the smaller micropipettes. Optimal diameter was 30 μm.
4. Depth of the 3D printed microwells. Optimal depth was 1 mm in order to efficiently trap the cells inside. A depth of 0.1 or 0.5 mm resulted in significantly more cells escaping from the wells.
5. Volume of the cell culture injected into the 5 × 5 mm^2^ wells. Optimal volume was 5 μl. Cells tended to escape from the well when using a higher volume or did not spread sufficiently in a smaller volume.
6. Diameter of the PDMS microwells was optimized for the cell size. Optimal diameter was 15 and 20 μm for human monocytes and 3T3 moue fibroblasts, respectively.

### Cell viability assay

We quantified the effect of the single cell isolation procedure on cell viability using 3T3 cells. Isolated cells were treated with trypan blue solution in a final concentration of 0.2% inside the miniature multiwell plate. After incubating cells with the trypan blue dye at room temperature for a few minutes, we captured images of all the isolated cells in both phase contrast and brightfield modes. We counted intact (not blue) and damaged (blue) cells. 95 ± 4% of the investigated n = 707 cells were viable after single cell isolation. Control cell viability was determined by placing 3T3 cells into untreated hydrophobic Petri dishes for 30 min at room temperature. Control cell viability was 94 ± 2% counting n = 594 cells.

### Pick up volume measurement

To calculate the volume of the culture medium in which single cells were isolated and then deposited, we measured the weight of the Petri dish on an analytical balance (Scaltec SBA 32) before and after repeating either the pickup process or the deposition process 1,000 times. Approximating the density of the culture medium with the density of water (1,000 kg/m^3^) the pickup volume was 1.4 ± 0.6 nl, while the deposition volume was 147 ± 11 nl. We kept the relatively high deposition volume to avoid the attachment of cells to the micropipette. Surface modification of the glass micropipette to inhibit cell adhesion is expected to allow to further decrease the deposition volume.

### Determination of the velocity of cell floating

We measured the speed of cell floating in the untreated hydrophobic 35 mm Petri dishes. 3T3 cells were treated with 1x trypsin-EDTA solution for 6 min at 37 °C, then the cell suspension was centrifuged at 300 g, for 2 min. In the first case cells were placed into the Petri dish in 2 ml cell culture medium. In the second case cells were pipetted into a thin (~100 μm) layer of culture medium covered by oil. In the third case cells were injected into a thin layer of medium under oil inside a 5 × 5 mm^2^ well of the miniature plate printed into the Petri dish. Phase contrast images were recorded on the microscope in every 30 s for 1 hour using the time-lapse module of the CellSorter software. We tracked manually the movement of 5–10 cells in each field of view. We calculated the average speed of cells tracked in 2–3 experiments for each case. We measured cell displacements in 120 s intervals (instead of 30 s), i.e., we averaged floating speed for a relatively long time of 2 min to decrease the error of the measurement. Speed of cell floating was diminished to 5.4 ± 1.1 μm/min in the thin layer of cell suspension covered by oil from the original 39 ± 12 μm/min measured in the usual medium height of 2 mm without an oil cover layer. Cell motility was further decreased to 2.3 ± 0.6 μm/min in the thin suspension when applying the 5 × 5 mm^2^ well (Fig. 1b).

## Additional Information

**How to cite this article**: Ungai-Salánki, R. *et al.* Automated single cell isolation from suspension with computer vision. *Sci. Rep.* **6**, 20375; doi: 10.1038/srep20375 (2016).

## Supplementary Material

Supplementary Information

## References

[b1] Shendure J. & Ji H. Next-generation DNA sequencing. Nat. Biotechnol. 26, 1135–1145 (2008). 18846087 10.1038/nbt1486

[b2] Navin N. *et al.* Tumour evolution inferred by single-cell sequencing. Nature 472, 90–94 (2011). 21399628 10.1038/nature09807 PMC4504184

[b3] Jaitin D. A. Massively Parallel Single-Cell RNA-Seq for Marker-Free Decomposition of Tissues into Cell Types. Science 343, 776–779 (2014). 24531970 10.1126/science.1247651 PMC4412462

[b4] Hempel C. M., Sugino K. & Nelson S. B. A manual method for the purification of fluorescently labeled neurons from the mammalian brain. Nature Protocols 2, 2924 (2007). 18007629 10.1038/nprot.2007.416

[b5] Herzenberg L. A., Sweet R. G. & Herzenberg L. A. Fluorescence-activated cell sorting. Sci. Am. 234, 108–117 (1976). 1251180 10.1038/scientificamerican0376-108

[b6] Herzenberg L. *et al.* The history and future of the fluorescence activated cell sorter and flow cytometry: a view from Stanford. Clin. Chem. 48, 1819–1827 (2002). 12324512

[b7] Fu A. Y. *et al.* A microfabricated fluorescence-activated cell sorter. Nat. Biotechnol. 17, 1061–1062 (1999). 10545919 10.1038/15095

[b8] Wolff A. *et al.* Integrating advanced functionality in a microfabricated high-throughput fluorescent-activated cell sorter. Lab Chip. 3, 22–27 (2003). 15100801 10.1039/b209333b

[b9] Emmert-Buck M. R. *et al.* Laser Capture Microdissection. Science 274, 998–1001 (1996). 8875945 10.1126/science.274.5289.998

[b10] Koller M. R. *et al.* High-throughput laser-mediated *in situ* cell purification with high purity and yield. Cytometry Part A 61A, 153–161 (2004). 10.1002/cyto.a.20079 15382147

[b11] Melin J. & Quake S. R. Microfluidic large-scale integration: the evolution of design rules for biological automation. Annual review of biophysics and biomolecular structure 36, 213 (2007). 10.1146/annurev.biophys.36.040306.132646 17269901

[b12] Jang J. S. *et al.* Quantitative miRNA Expression Analysis Using Fluidigm Microfluidics Dynamic Arrays. BMC Genomics 12, 144 (2011). 21388556 10.1186/1471-2164-12-144 PMC3062620

[b13] Hosokawa M. *et al.* High-Density Microcavity Array for Cell Detection: Single-Cell Analysis of Hematopoietic Stem Cells in Peripheral Blood Mononuclear Cells. Anal Chem. 81, 5308 (2009). 19485404 10.1021/ac900535h

[b14] Schneider A. *et al.* “The Good into the Pot, the Bad into the Crop!”—A New Technology to Free Stem Cells from Feeder Cells. PLoS ONE 3, e3788 (2008). 19023443 10.1371/journal.pone.0003788 PMC2582950

[b15] Környei Z. *et al.* Cell sorting in a Petri dish controlled by computer vision. Sci Rep. 3, Article number: 1088 (2013). 10.1038/srep01088 PMC3548191 23336070

[b16] Salánki R. *et al.* Automated single cell sorting and deposition in submicroliter drops. Appl. Phys. Lett. 105, 083703 (2014).

[b17] Yoshimoto N. *et al.* An automated system for high-throughput single cell-based breeding. Sci Rep. 3, 1191 (2013). 23378922 10.1038/srep01191 PMC3561619

[b18] Zhu Y. *et al.* Printing 2-dimentional droplet array for single-cell reverse transcription quantitative PCR assay with a microfluidic robot. Sci. Rep. 5, 9551 (2015). 25828383 10.1038/srep09551 PMC4381353

[b19] Anis Y. H., Holl M. R. & Meldrum D. R. Automated selection and placement of single cells using vision-based feedback control. IEEE Transactions on Automation Science and Engineering 7, 598 (2010).

[b20] Swennenhuis J. F. *et al.* Self-seeding microwell chip for the isolation and characterization of single cells. Lab Chip 15, 3039–46 doi: 10.1039/C5LC00304K (2015). 26082273

[b21] Guo M. T., Rotem A., Heyman J. A. & Weitz D. A. Droplet microfluidics for high-throughput biological assays. Lab Chip 12, 2146–2155 (2012). 22318506 10.1039/c2lc21147e

[b22] Clausell-Tormos J. *et al.* Droplet-Based Microfluidic Platforms for the Encapsulation and Screening of Mammalian Cells and Multicellular Organisms. Chemistry & Biology 15, 427–437 (2008). 18482695 10.1016/j.chembiol.2008.04.004

[b23] Rettig J. R. & Folch A. Large-scale single-cell trapping and imaging using microwell arrays. Anal. Chem. 77, 5628–5634 (2005). 16131075 10.1021/ac0505977

[b24] Qin D., Xia Y. & Whitesides G. M. Soft lithography for micro- and nanoscale patterning. Nature Protocols 5, 495–502 (2010). 10.1038/nprot.2009.234 20203666

[b25] del Campo A. & Greiner C. SU-8: a photoresist for high-aspect-ratio and 3D submicron lithography – TOPICAL REVIEW. J.Micromech. Microeng. 17, R81–R95 (2007).

[b26] Mata A., Fleischman A. J. & Roy S. Fabrication of multi-layer SU-8 microstructures. J. Micromech. Microeng. 16, 276–28 (2006).

[b27] Rasband W. ImageJ Image Processing and Analysis in Java. http://imagej.nih.gov/ij (Date of access:02/12/2015).(1997).

