# Supplementary material for: Automated single cell isolation from suspension with computer vision
Source: Sci Rep. 2016 Feb 9;6:20375. doi: 10.1038/srep20375 (PMC4746594; doi:10.1038/srep20375)
Supplement: Supplementary Information [file srep20375-s1.pdf]

Automated single cell isolation from suspension with computer vision

Supplementary Information

Rita Ungai-Salánki [1,2,3], Tamás Gerecsei [3], Péter Fürjes [4], Norbert Orgovan [2,3], Noémi Sándor [5], Eszter Holczer [4], Robert Horvath [2], Bálint Szabó [2,3,6] *

[1]*Doctoral School of Molecular- and Nanotechnologies, University of Pannonia, Veszprém, Hungary*
[2]*Nanobiosensorics Group, Institute of Technical Physics and Materials Science, Centre for Energy Research, Hung. Acad. Sci., Budapest, Hungary*
[3]*Department of Biological Physics, Eötvös University, Pázmány Péter sétány 1A, Budapest, H-1117 Hungary*
[4]*MEMS Lab, Institute of Technical Physics and Materials Science, Centre for Energy Research, Hung. Acad. Sci., Budapest, Hungary*
[5]*MTA-ELTE Immunology Research Group, Budapest, Hungary*
[6] *CellSorter Company for Innovations, Budapest, Hungary*

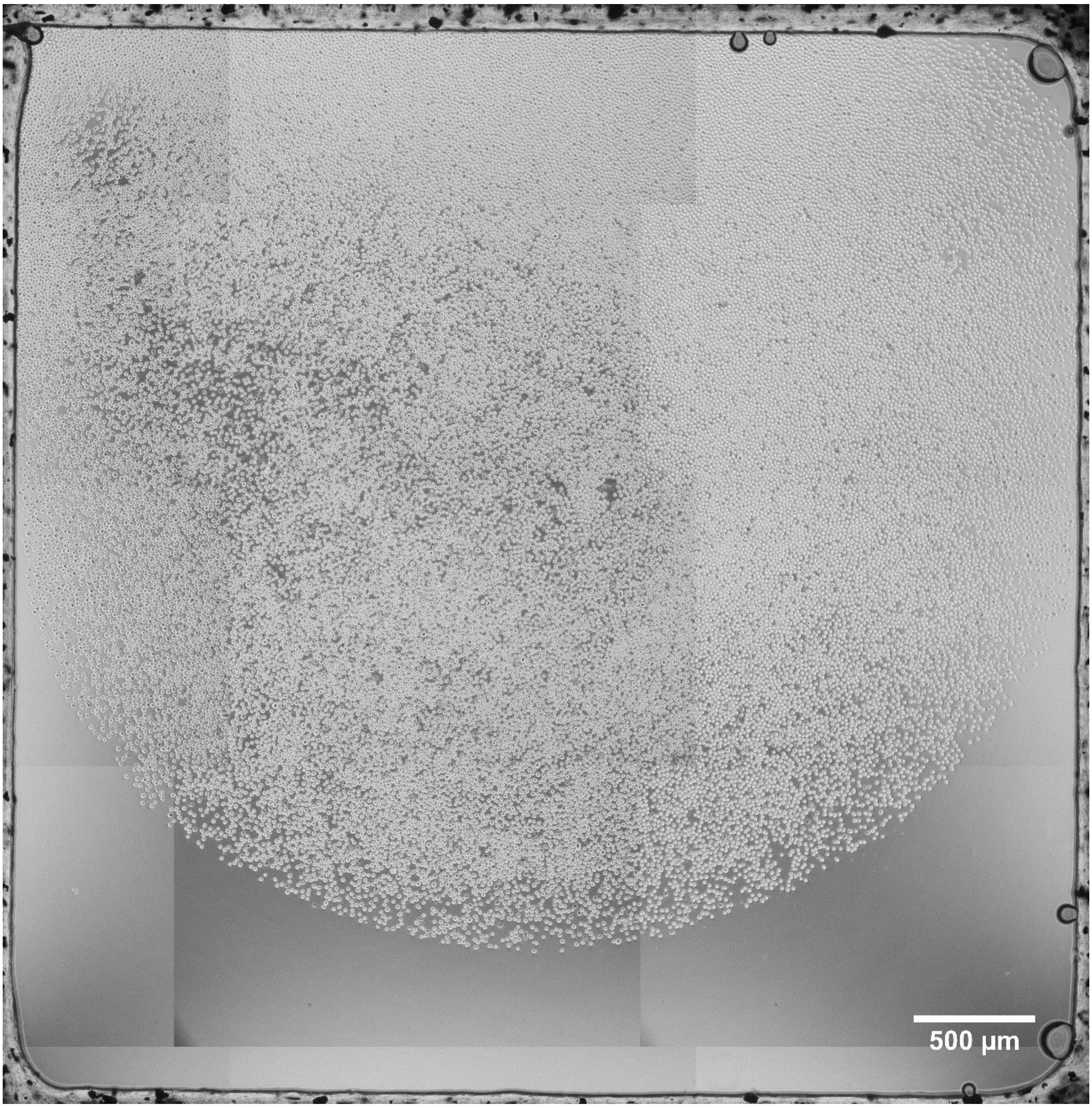

Supplementary Figure S1. Phase contrast mosaic image of a 5x5 mm^2 square printed into a Petri dish containing 50,000 cells. Cells were injected in a volume of 5 µl into the thin (~100 µm) layer of culture medium under the oil cover layer using a standard laboratory pipette. Walls of the square with a height of 1 mm could keep more than 99 % of cells inside.

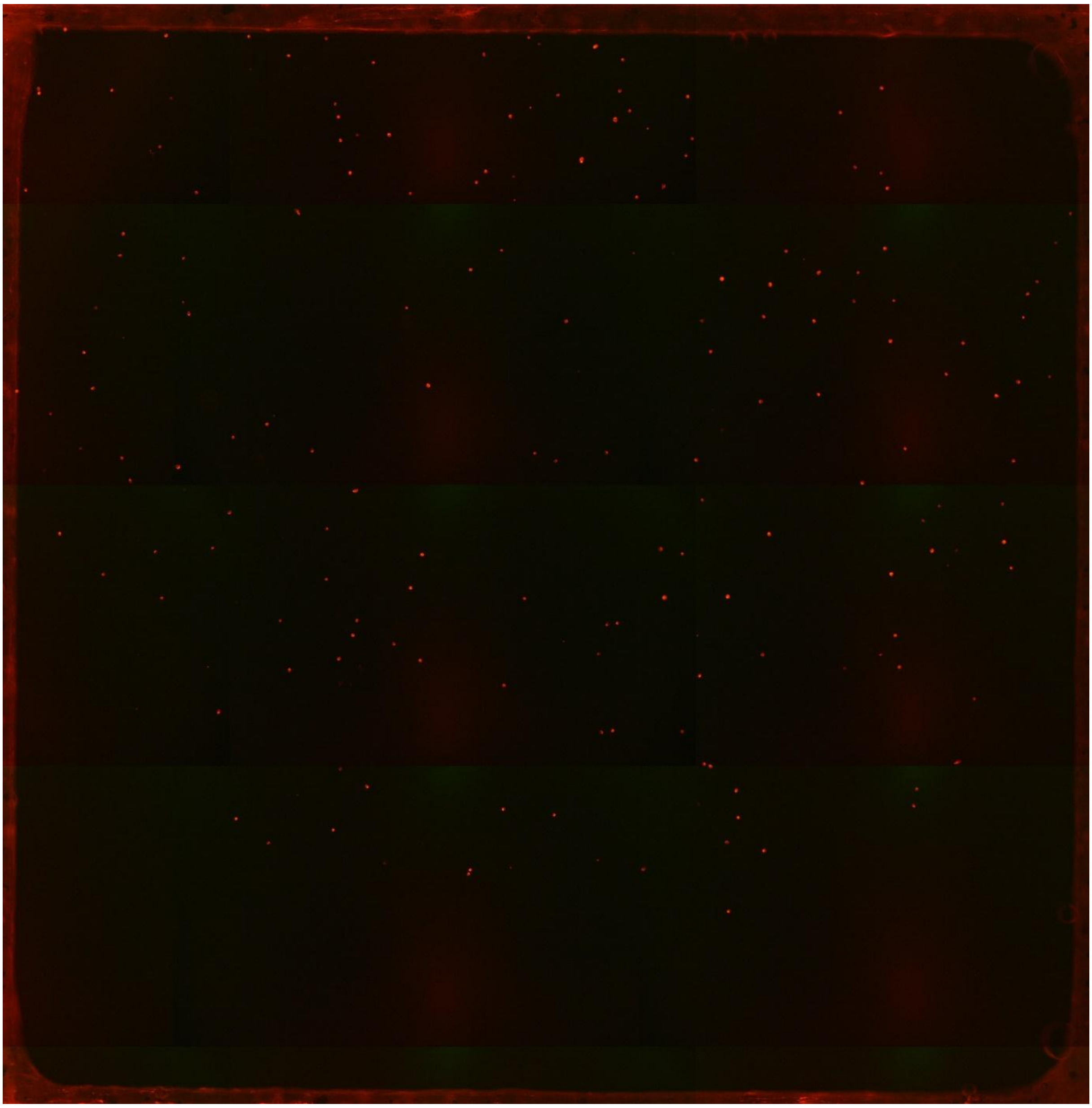

Supplementary Figure S2. Same region as shown in **Supplementary Fig. 1** but captured in fluorescent mode. The 179 labeled cells can be seen in red.

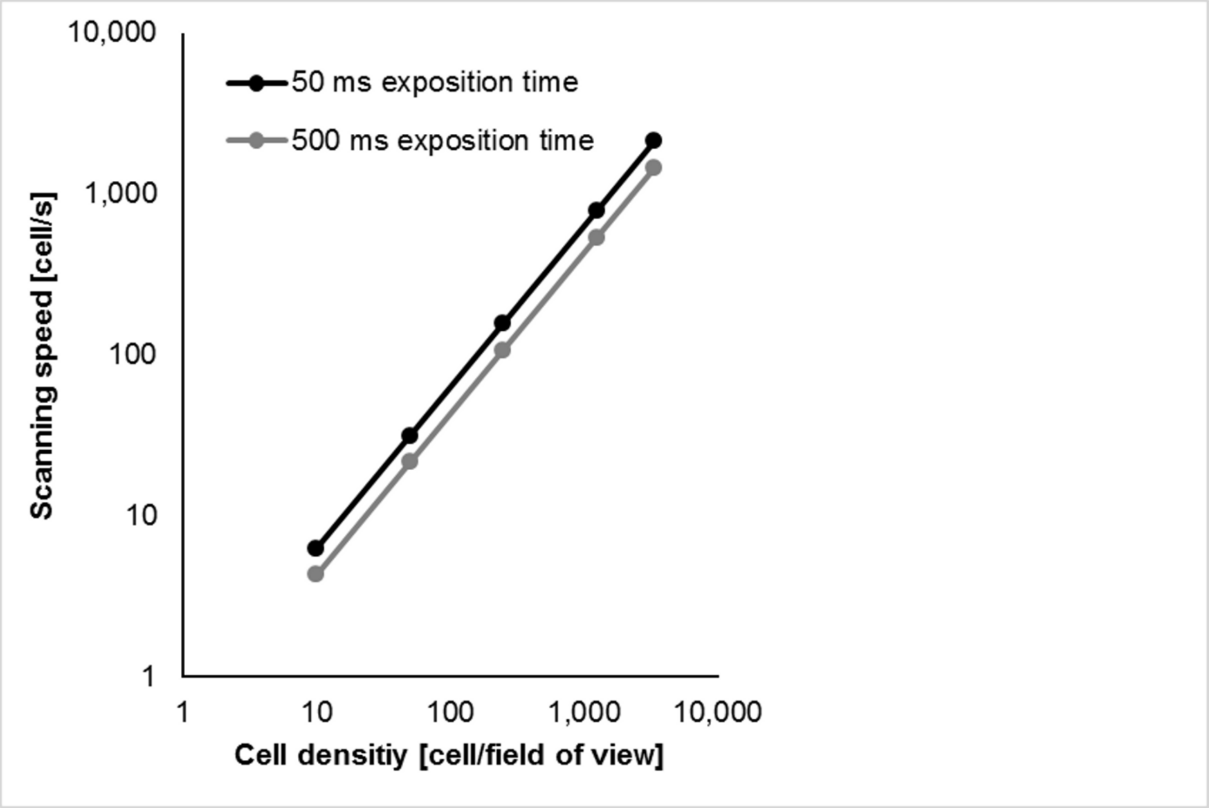

Supplementary Figure S3. Scanning speed as a function of cell density. Scanning speed of the microscope was limited by the mechanics of the motorized stage and the exposition time of the camera. For the scanning speed measurement we used the 10x objective lens and an Andor Zyla 5.5 USB 3 camera with a resolution of 4 Megapixel. We decreased the acceleration of the motorized stage to 1% in the CellSorter software to avoid shaking the cell suspension. Maximum cell density was 2,000 cell/mm^2 (3,400 cell/field of view).

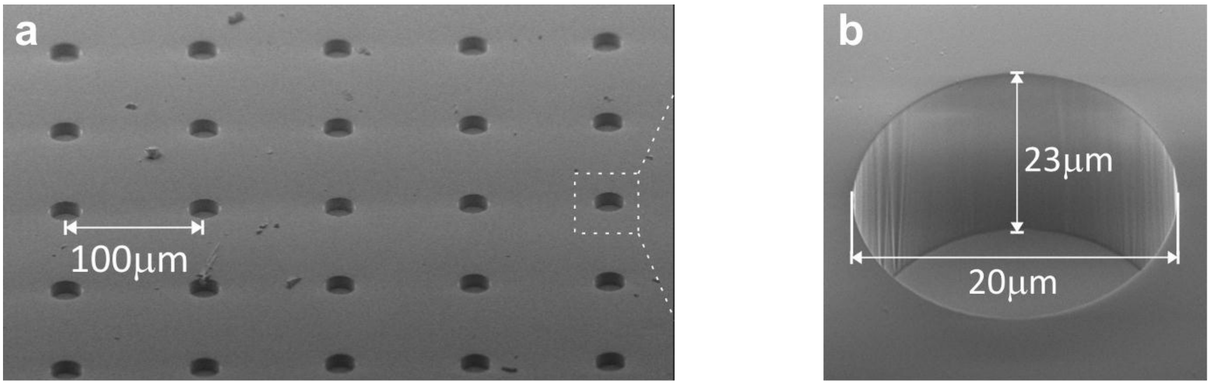

Supplementary Figure S4. Scanning electron micrograph of the PDMS microwell array developed by soft lithography (**a**) and a zoomed in image of a microwell (**b**). We used wells with a diameter of 15 and 20 μm fitted to the size of human monocytes and 3T3 cells, respectively. As the spatial resolution of the I.D. 70 μm micropipette normally used to pick surface attached cells is ~70 μm[10,11], we applied microwells arranged into a grid with a lattice constant of 100 μm to avoid picking unselected cells from the neighbouring wells.

	**Number of cells selected**	**Number of single cells**	**Number of double cells**	**Number of cells not picked**	**Single cell pick up efficiency (%)**
**Sorting from microwells**					
**3T3 cells**	23	18	0	5	78
	30	30	0	0	100
	58	23	4	35	40
	28	16	0	12	57
	26	11	0	15	42
	33	10	0	23	30
	34	7	0	27	21
**Total**	232	115	4	117	**50 ± 10 %**
**Monocytes**					
	98	71	0	27	72
	82	34	0	48	41
	49	11	0	38	22
**Total**	229	116	0	113	**51 ± 14 %**
**Sorting from a thin layer of suspension**					
**3T3 cells**	17	12	2	3	71
	14	12	2	0	86
	21	15	6	0	71
	11	8	1	2	73
	8	8	0	0	100
	37	32	2	4	86
	27	18	1	8	67
**Total**	135	105	13	17	**78 ± 4 %**
**Monocytes**					
	28	20	3	5	71
	17	15	1	1	88
	17	12	0	5	71
	28	20	6	1	71
	18	14	4	0	78
**Total**	108	81	14	12	**75 ± 3 %**

Supplementary Table S1. Comparison of the efficiency of single cell isolation from a sparse cell suspension to that from PDMS microwells. Single cell sorting efficiency of 3T3 cells and human monocytes were improved to 78 ± 4 % from 50 ± 10 % and to 75 ± 3 % from 51 ± 14 %, respectively, when sorting from suspension instead of using microwells.
